# Complement 5a‐mediated trophoblasts dysfunction is involved in the development of pre‐eclampsia

**DOI:** 10.1111/jcmm.13466

**Published:** 2017-11-23

**Authors:** Yu Ma, Ling‐Ran Kong, Qian Ge, Yuan‐Yuan Lu, Mo‐Na Hong, Yu Zhang, Cheng‐Chao Ruan, Ping‐Jin Gao

**Affiliations:** ^1^ State Key Laboratory of Medical Genomics Shanghai Key Laboratory of Hypertension Department of Hypertension Ruijin Hospital and Shanghai Institute of Hypertension Shanghai Jiao Tong University School of Medicine Shanghai China; ^2^ Department of Obstetrics and Gynecology Ren Ji Hospital Shanghai Jiao Tong University School of Medicine Shanghai China; ^3^ Laboratory of Vascular Biology and Key Laboratory of Stem Cell Biology Institute of Health Sciences Shanghai Institutes for Biological Sciences Chinese Academy of Sciences & Shanghai Jiao Tong University School of Medicine Shanghai China

**Keywords:** pre‐eclampsia, complement 5a, C5a receptor, placenta, trophoblasts, angiogenesis, arterial stiffness

## Abstract

Pre‐eclampsia (PE) is a life‐threatening multisystem disorder leading to maternal and neonatal mortality and morbidity. Emerging evidence showed that activation of the complement system is implicated in the pathological processes of PE. However, little is known about the detailed cellular and molecular mechanism of complement activation in the development of PE. In this study, we reported that complement 5a (C5a) plays a pivotal role in aberrant placentation, which is essential for the onset of PE. We detected an elevated C5a deposition in macrophages and C5a receptor (C5aR) expression in trophoblasts of pre‐eclamptic placentas. Further study showed that C5a stimulated trophoblasts towards an anti‐angiogenic phenotype by mediating the imbalance of angiogenic factors such as soluble fms‐like tyrosine kinase 1 (sFlt1) and placental growth factor (PIGF). Additionally, C5a inhibited the migration and tube formation of trophoblasts, while, C5aR knockdown with siRNA rescued migration and tube formation abilities. We also found that maternal C5a serum level was increased in women with PE and was positively correlated with maternal blood pressure and arterial stiffness. These results demonstrated that the placental C5a/C5aR pathway contributed to the development of PE by regulating placental trophoblasts dysfunctions, suggesting that C5a may be a novel therapeutic possibility for the disease.

## Introduction

PE, characterized by new‐onset hypertension and proteinuria, is a severe obstetric complication leading to maternal and foetal morbidity and mortality 1, 2. Although PE is well known as a “pregnancy‐specific” syndrome, women who develop this disorder are more vulnerable to long‐term cardiovascular consequences in later life 3, 4, 5. Despite the complicated etiopathogenesis, it is generally agreed that the placenta is the central organ in the pathogenesis of PE because the only current ‘cure’ for the disorder is to deliver the placenta 6. Abnormal trophoblast function and poor uterine spiral artery remodelling are hallmarks of aberrant placentation, a process associated with the development of PE 7, 8, 9. However, the cellular and molecular mechanisms underlying PE are still unclear.

The immune system is thought to play a pivotal role in the pathophysiology of PE. Women who have autoimmune diseases, such as systemic lupus erythematosus or anti‐phospholipid syndrome, are more likely to develop PE 10, 11, indicating the association of immune responses and PE. The complement system is an integral component of the innate immune system and regulates the adaptive immune system as well 12. Growing evidence suggests that complement system plays a vital role in the pathogenesis of PE. Previous study showed that complement C1q prevents the development of PE through regulating placental oxidative stress and angiogenic molecule expression. Pregnant C1q‐deficient mice recapitulate the key features of human PE including hypertension and albuminuria 13. In contrast, complement C3 appeared to mediate adverse pregnancy outcomes. Wang *et al*. reported that angiotensin II type 1 receptor agonistic autoantibody contributes to C3 and C3aR signalling is a key mechanism underlying the pathogenesis of PE 14. As a key downstream component of the complement cascade, C5a exerts a pro‐inflammatory effect by binding to its respective receptor, C5aR 15. Our previous study showed that C5a, the major production from C3 activation, contributes to vascular injury in hypertensive model 16. Yet, whether C5a could regulate placental vascular remodelling is not clear. It has been reported that plasma levels of C5a were increased in women with PE 17. However, the resident distribution of C5a in placentas and the detailed cellular and molecular mechanism of the C5a/C5aR pathway in PE are still unknown. Thus, we investigated the effect of the C5a/C5aR pathway on trophoblasts and the association between C5a and maternal arterial stiffness.

## Materials and methods

### Ethics statement

This study was approved by the Ethics Committee of Ruijin Hospital, Shanghai Jiao Tong University School of Medicine, China, and conducted in accordance with the Declaration of Helsinki. Signed informed consent was obtained from all study participants.

### Tissue collection

Placenta tissues were obtained from women (PE group and normal pregnant group, *n *=* *6 per group) who were hospitalized in the Department of Gynecology and Obstetrics of Shanghai Jiao Tong University School of Medicine. Placenta tissue blocks (1 cm^3^) from central parts of the maternal side were collected after the placentas delivered, and immediately washed in ice‐cold phosphate‐buffered saline (PBS) 2 or 3 times to clear the blood, then placed in liquid nitrogen or 4% paraformaldehyde for further study.

### Animal model

Pregnant mice were given Nω‐nitro‐l‐argininemethyl ester hydrochloride (L‐NAME, a NOS inhibitor; Sigma‐Aldrich N5751, St. Louis, MO, USA) in drinking water (100 mg/kg/d (1 g/l)) at gestational day 8 (gd 8) to mimic the PE‐like symptoms. Mice were killed at day 12 and day 20, and the placentas were collected for further study.

### Western blot

Western blot analysis was performed as previously described 16. Primary antibodies were C5a (Comp Tech, Tyler, TX, USA), C5aR (Biolegend, San Diego, CA, USA) and GAPDH (PTG, USA). Secondary antibodies were obtained from Santa Cruz Biotechnology (Dallas, TX, USA). GAPDH was used to normalize for loading variability. All experiments were performed with at least three replications.

### Histology and immunofluorescence staining

The placentas were fixed in 4% paraformaldehyde and were subsequently embedded in paraffin. Then the 5‐μm‐thick sections were processed and stained with haematoxylin and eosin (HE, Maixin, Fuzhou, China). Immunofluorescence analysis was performed as previously described 16. The human placenta slices were blocked in 10% normal goat serum for 30 min., then incubated with primary antibodies against CD31 (Santa Cruz), CD11b (BD Biosciences, San Jose, CA, USA), C5a (Comp Tech, A221), C5aR (Biolegend), cytokeratin 7 (Cy7, Santa Cruz), PIGF (Proteintech group, Wuhan, Hubei, China), sFlt1 (Life Technologies, Waltham, MA, USA), IL‐1β (Boster), IL‐6 (Boster) and MCP‐1 (Boster, Wuhan, Hubei, China) at 4°C overnight. HTR‐8/SVneo cells were stained by immunofluorescence on coverslips. Briefly, cells were washed with phosphate‐buffered saline (PBS), fixed in 4% paraformaldehyde for 20 min., and permeabilized with 0.25% Triton X‐100 for 10 min., followed by incubation with PIGF and sFlt1 primary antibodies. Subsequently, sections and coverslips were stained with fluorescent secondary antibodies. Nuclei were stained with DAPI. Sections were observed and imaged using a laser scanning confocal microscope (LSM 710; Carl Zeiss, Germany).

### Cell proliferation

Human extravillous trophoblast cell line HTR‐8/Svneo (obtained from ATCC) was incubated at 37°C with 5% CO_2_. C5aR‐specific small interfering RNA (siRNA, Santa Cruz) was transfected into HTR‐8/SVneo cells using Lipofectamine 2000 (Invitrogen, Waltham, MA, USA), according to the manufacturer's instructions. A cell counting kit‐8 (Dojindo Molecular Technologies, Kumamoto, Japan) was used to evaluate the effect of C5a on trophoblast cell viability as the manufacturer's protocol. In brief, HTR‐8/SVneo cells were seeded in 96‐well plates at a density of 5 × 10^3^ cells/well. After incubation for 24 hrs at 37°C, cells were starved for 6 hrs in serum‐free medium, and then treated with C5a (human C5a peptide agonist, which can bind to its respective receptor, will be represented as C5a throughout the paper) at different concentration (0, 25, 50, 100 nM) for 24 or 48 hrs. Subsequently, 10 μl CCK‐8 solution (10%) was added to each well and incubated for another 1 hr. The absorbance was measured at 450 nm using a microplate reader (ThermoFisher, Waltham, MA, USA).

### Trophoblasts transwell migration assay

*In vitro*, migration of HTR‐8/SVneo cells was assessed by the transwell migration chambers (24‐well inserts, 8.0 μm pores, Corning Costar, Cambridge, MA, USA). Cells were pre‐treated with C5a (100 nM) for 24 hrs. Then, 500 μl DMEM/F12 medium containing 10% FBS was added to the lower chamber, and 1 × 10^5^ cells suspended in 100 μl serum‐free DMEM/F12 with 100 nM of C5a (and PBS as control) were seeded into the upper chamber respectively. After incubating for 24 hrs, the chambers were fixed with 4% paraformaldehyde and stained with 0.1% crystal violet. The number of migrated cells attached to the other side of the upper chamber was counted under the microscope (10 random fields per well). Results are expressed as mean ± S.E.M.

### HTR‐8/SVneo capillary‐like tube formation assay

The tube formation assay was performed to examine the effect of C5a on angiogenesis of HTR‐8/SVneo cells. BD Matrigel was thawed at 4°C overnight, and the 96‐well plates and pipette tips were pre‐cooled. 50 μl Matrigel was added into each well of the 96‐well plate, and then the plate was incubated for at least 30 min. at 37°C. After the Matrigel solidified, HTR‐8/SVneo cells (1.5 × 10^4^ per well) with C5a peptide (100 nM) or PBS were added on the top of the gel. After incubation for 6 hrs, the capillary‐like tube formation was observed, and the images were taken from at least five randomly selected fields per well. Quantification of the tubular network was measured by the Image Pro Plus software.

### RNA isolation and quantitative real‐time PCR

Total RNA was isolated from placentas or cultured cell lines using TRIzol reagent (T9424, Sigma‐Aldrich) according to the manufacturer's instructions. After RNA, concentrations and quality were detected on a NanoDrop spectrophotometer (ThermoFisher, USA), 1 μg total RNA was reverse transcribed into cDNA using Tanscriptor First Strand cDNA Synthesis Kit (Roche, Mannheim, Germany). Quantitative real‐time PCR was performed on Viia 7 Real‐Time PCR System and StepOne Plus System (Applied Biosystems, Foster City, CA, USA) with the Faststart Universal SYBR Green Master (Roche). The following PCR cycle was conducted: 10 min. at 95°C and 40 cycles, each cycle containing 10 sec. at 95°C, 30 sec. at 60°C. The value of each sample was normalized to the β‐actin mRNA expression. Sequences of the primers were listed Table S1.

### Study subjects

We recruited 56 women in total: 24 women with established PE and 32 normal pregnant women. PE was defined as hypertension (blood pressure of 140 mmHg systolic or higher or 90 mmHg diastolic or higher occurred after 20 weeks of gestation in previously normotensive women) with proteinuria (≥300 mg total protein in a 24‐hrs urine collection or two readings of dipstick measurement of 1+ or more) according to the American College of Obstetricians and Gynecologists guidelines 18. Women with diabetes mellitus, cardiovascular disease, renal disease, autoimmune disease, malignancies or women who had recent trauma or surgery were excluded. Normal pregnant women were selected on the basis of having a normotensive and uncomplicated pregnancy with a normal term delivery. Maternal age, gestational weeks at entry, gestational weeks at delivery, parity, body mass index (BMI), blood pressure (BP) and birthweight were recorded.

### Measurement of C3a and C5a

Blood samples were collected using BD Vacutainer® tubes. After centrifugation at 3000 r.p.m. for 10 min. at 4°C, serum specimens were separated from the clot. The supernatant obtained was then aliquoted and kept at −80°C until analysed. Serum concentrations of C3a and C5a were detected with C3a and C5a ELISA kit (BD Biosciences), respectively. The samples were assayed according to the manufacturer's instructions.

### Arterial stiffness and wave reflection measurement

The pulse wave analysis was measured by a high‐fidelity SPC‐301 micromanometer (Millar Instruments, Houston, TX, USA) interfaced with a laptop computer running the SphygmoCor software version 7.1 (AtCor Medical, West Ryde, Australia) as previously described 19. From the radial signal, the SphygmoCor software calculates the aortic pulse wave by means of a validated generalized transfer function. The central (aortic) systolic and diastolic blood pressures were derived from the aortic pulse wave. The AIx was the ratio of the second to the first peak of the pressure wave expressed in percentage. AIx values were automatically recalculated and standardized to a heart rate of 75 beats per minute. For the measurement of carotid–femoral pulse wave velocity (cf‐PWV), the operator recorded in succession the right carotid and femoral waveforms (12 sec. each). With the simultaneously recorded electrocardiogram (lead 2), the time delay between the rapid upstroke of the right common carotid artery and the right femoral artery was measured. The distance travelled by the pulse wave was estimated by measuring the distance between the two recording sites over the body surface with a tape measure. PWV was calculated as the distance travelled divided by the transit time.

Brachial–ankle pulse wave velocity (ba‐PWV) was performed using Vascular Profiler‐1000 device (Omron, Kyoto, Japan) as previously described and validated 20, 21. The subjects were examined in the supine position after at least 10 min. of rest. The device then simultaneously measured bilateral brachial and posterior tibial arterial pulse volume waveforms and BP using an oscillometric cuff technique. The transmission distance was calculated automatically according to the height of the subject. The ba‐PWV was measured as the ratio of transmission distance divided by the time interval between the pulse waveforms transmitted from brachial to ankle arteries. The higher value of ba‐PWV between the right and left sides was used for the analyses.

### Statistical analysis

All statistical analysis was carried out with SPSS version 20.0 and Graphpad Prism 5. Student's *t*‐test was used to analyse the normally distributed continuous variables and Mann–Whitney *U*‐test was used to analyse the skewed variables. All values were presented as mean ± S.E.M. or median (interquartile range). For determination of potential predictors of PE, a univariate logistic regression analysis was used to evaluate the predictive power of each variable. The variables that were statistically significant in the univariate regression analysis were subsequently tested in a multiple logistic regression model. The odds ratios (OR) with their confidence intervals (CI) of 95% are presented. Correlations between variables were calculated with Pearson correlation coefficient. Longitudinal data were analysed by repeated measures anova with Bonferroni post hoc test for pairwise comparisons. A *P*‐value < 0.05 was considered statistically significant.

## Results

### Histological findings

**Figure 1:**
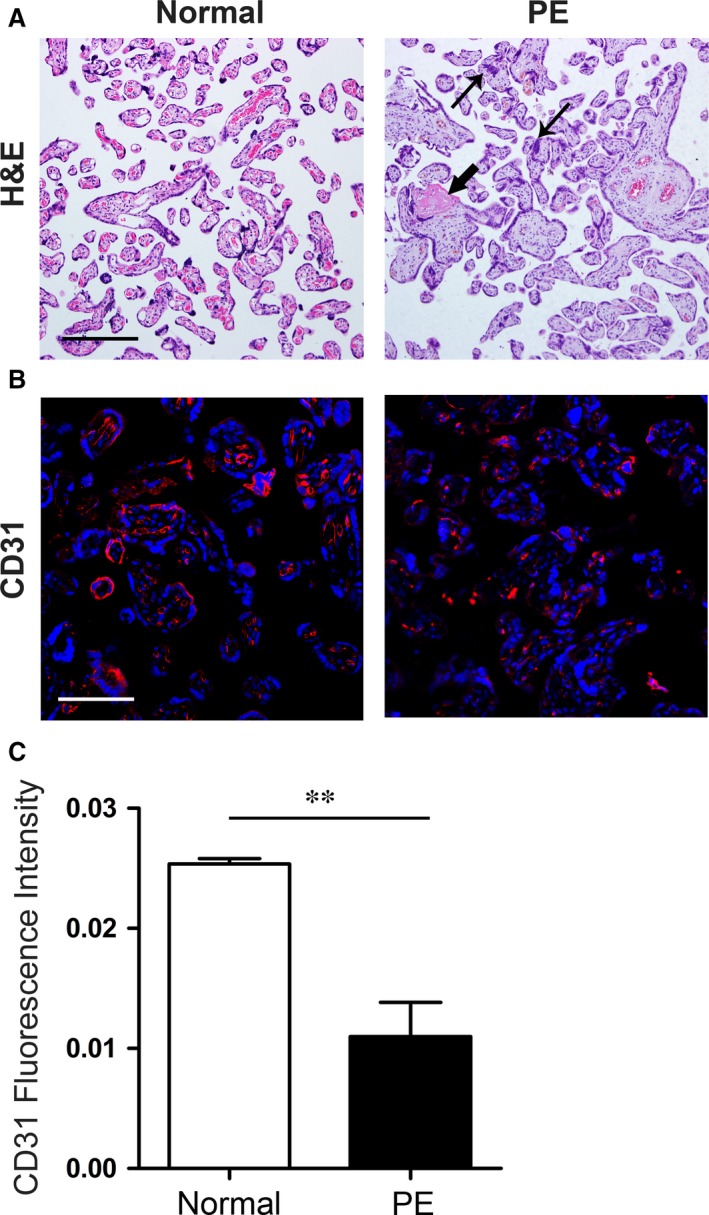
Histopathologic abnormalities in placentas of PE group (**A**) HE staining of placental sections from women with PE and compared to normal pregnant women shows an increased level of fibrinoid necrosis (thick arrow) and syncytial knot (thin arrow). (**B**) Immunofluorescence staining showed decreased expression of CD31 (red, endothelial cell marker) in placentas of women with PE. Scale bar: 100 μm. (**C**) Statistical data of CD31 staining. *N *=* *6 in each group. Data are shown as mean ± S.E.M.; **P <* *0.01.

Compared to the placentas in the control group, the placentas in the PE group had increased fibrinoid necrosis and syncytial knot (a multinucleated aggregate of syncytial nuclei at the surface of terminal villi in the placenta) formation (Fig. 1A). Expression of CD31 (endothelial cell marker) was significantly decreased in placentas of PE group (Fig. 1B, C). The data revealed that placentas of women with PE displayed histopathologic abnormalities and poor placentation.

### Expression of C5a and C5aR in placentas

**Figure 2:**
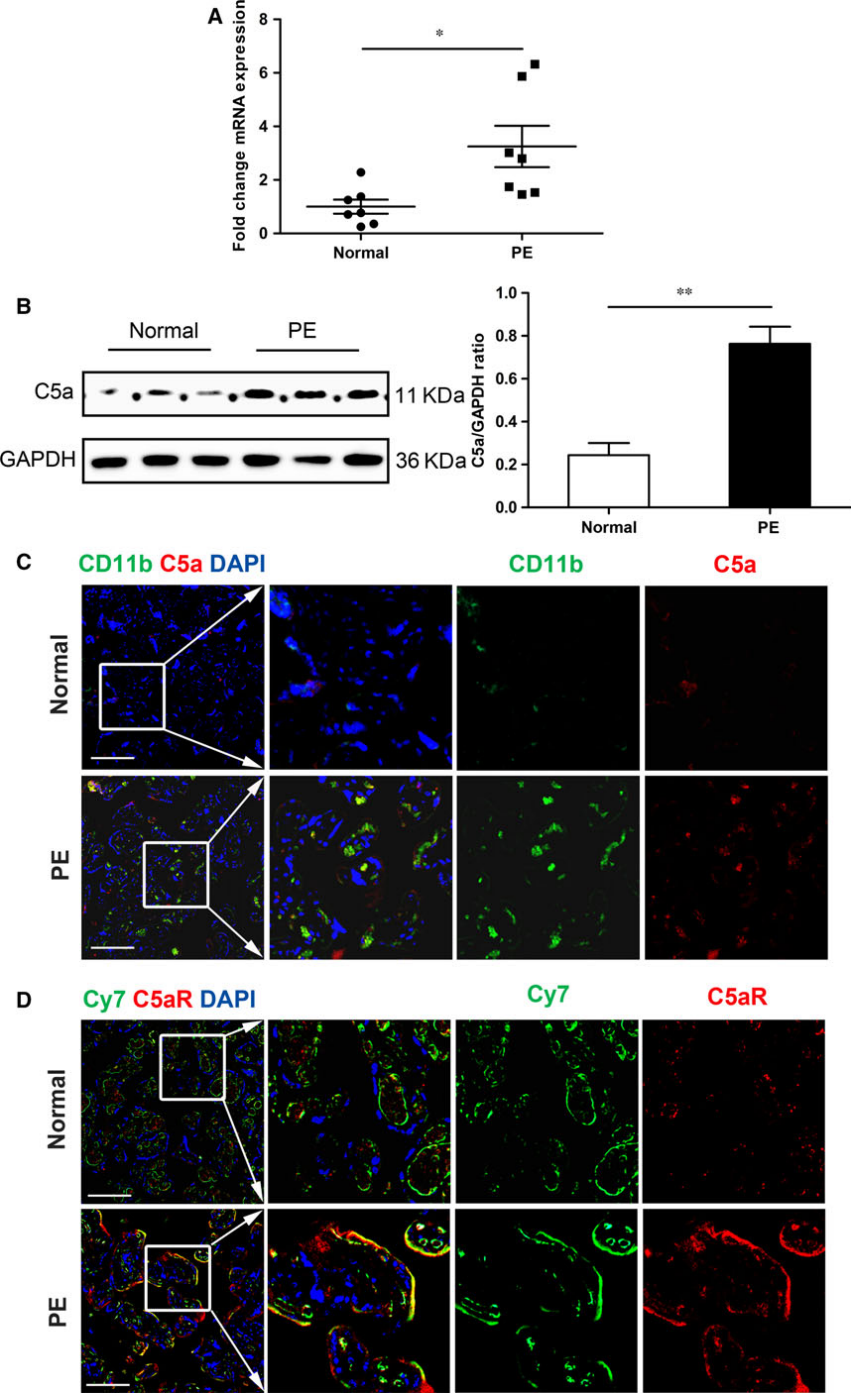
Expression of complement in maternal circulation and placentas. (**A**) C5 mRNA levels in human placentas of normal pregnant women and women with PE. Data are shown as mean ± S.E.M., *P <* *0.05. (**B**) Western blot and quantification of C5a protein expression in placentas of two groups. Data are shown as mean ± S.E.M., **P <* *0.01. (**C**) Immunofluorescence analysis of CD11b^+^ macrophages (green) and C5a (red) expression in human placenta. Scale bar: 100 μm. (**D**) Colocalization of C5aR (red) with Cy7 (green, trophoblast cells marker) in human placentas. Nuclei were counterstained with DAPI (blue). Scale bar: 100 μm.

Placental C5a level was increased in mice placentas in early pregnancy with L‐NAME treatment and further enhanced in late pregnancy (Fig. S1). We also detected the expression of C5a in pre‐eclamptic and normal pregnant women. C5 mRNA level and C5a protein level in the placentas were significantly higher in PE group compared with normal pregnant group (*P *<* *0.05, mean ± S.E.M.; Fig. 2A, B). Furthermore, immunofluorescence staining showed that the expression of C5a was dramatically enhanced in pre‐eclamptic placentas and was colocalized with CD11b^+^ macrophages (Fig. 2C). We also found that C5aR was mainly colocalized with syncytiotrophoblast and cytotrophoblast (Fig. 2D). *In vitro*, we further demonstrated that C5a was expressed in macrophages (Thp‐1 cell) and C5aR was dominantly expressed on trophoblasts (Fig. S2). The data suggest a potential link between C5a/C5aR pathway and trophoblasts dysfunction in the PE.

### Effect of C5a on generation of angiogenic factors in trophoblasts

**Figure 3:**
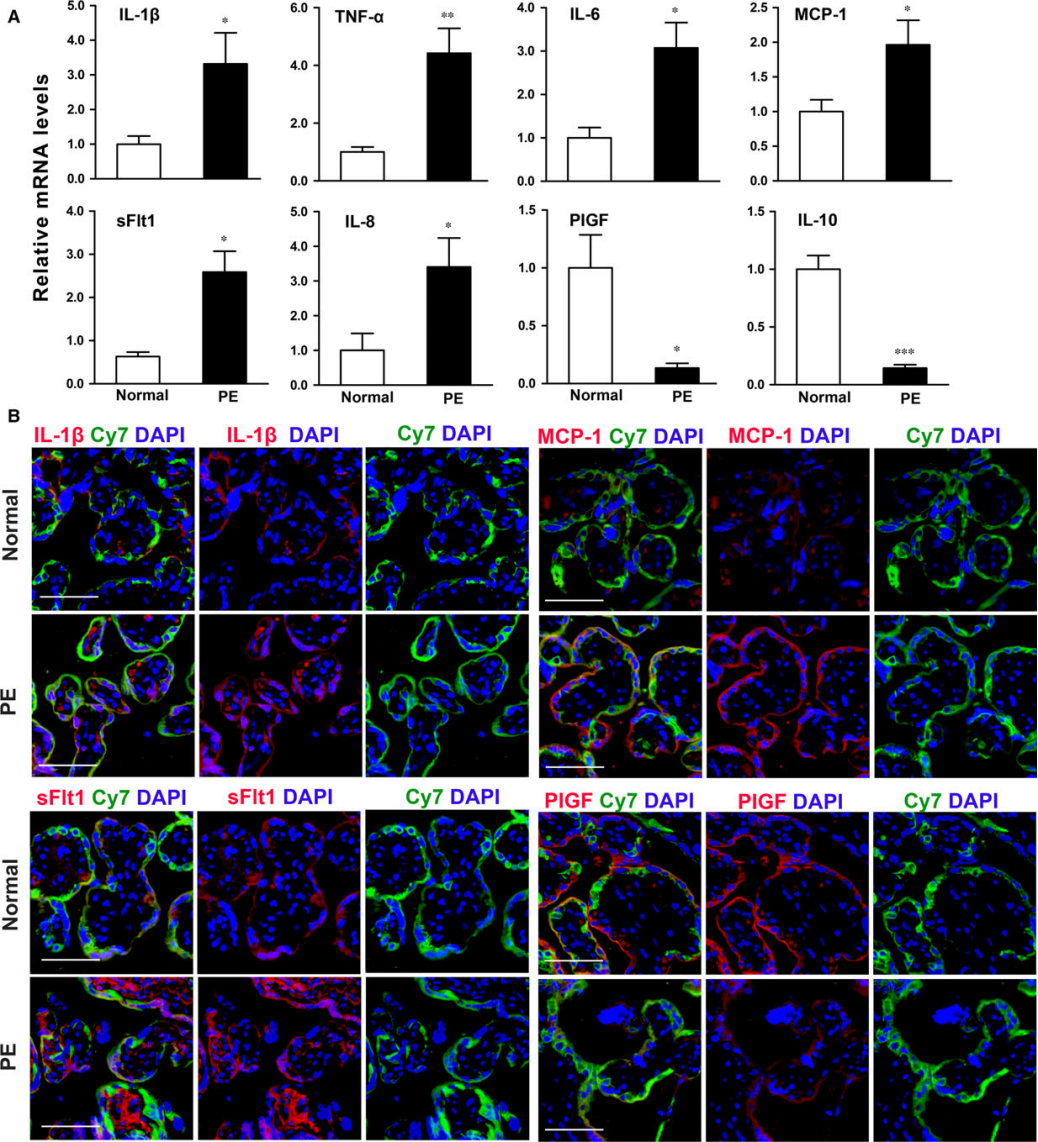
Expression of angiogenesis‐related factors in human placenta. (**A**) Relative mRNA levels of anti‐angiogenic factors (IL‐1β, TNF‐α, IL‐6, MCP‐1 and sFlt1) and pro‐angiogenic (PIGF, IL‐10) in normal and pre‐eclamptic placentas. Data are represented as mean ± S.E.M. *N *=* *4–6 in each group. *P <* *0.05, **P <* *0.01, ***P <* *0.001. (**B**) Immunofluorescence analysis of representative angiogenesis‐related factors (red) and Cy7 (green) in normal and PE placentas. Nuclei were counterstained with DAPI (blue). Scale bar: 60 μm.

The imbalances of placenta‐released angiogenic factors contribute to placental dysfunction, leading to the onset of PE. Therefore, we detected the expression of placental pro‐angiogenic and anti‐angiogenic factors in PE and normal pregnant groups. The mRNA levels of IL‐1β, TNF‐α, IL‐6, MCP‐1, sFlt1 and IL‐8 were significantly increased, and mRNA levels of PIGF and IL‐10 were dramatically decreased in women with PE as compared to normal controls (Fig. 3A). Immunofluorescence staining further confirmed that expressions of IL‐1β, MCP‐1 and sFlt1 were higher but PIGF was lower in pre‐eclamptic placentas compared to the normal controls (Fig. 3B).

**Figure 4:**
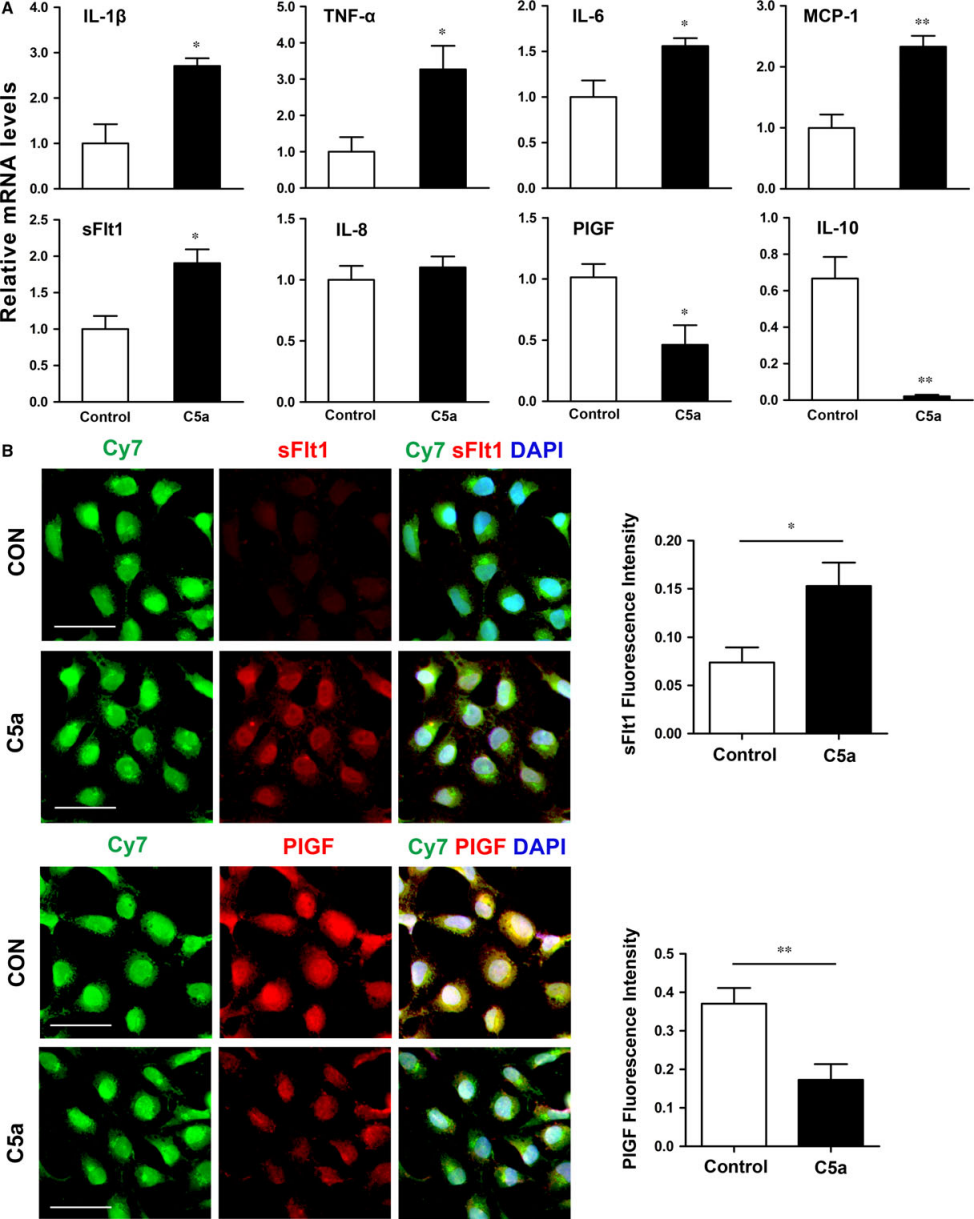
Trophoblasts stimulated with C5a display an anti‐angiogenic phenotype. (**A**) HTR‐8/SVneo cells treated with C5a showed a polarization towards an anti‐angiogenic phenotype with significantly increased mRNA levels of IL‐1β, TNF‐α, IL‐6, MCP‐1, sFlt1 and decreased mRNA level of PIGF and IL‐10. The respective mRNA was normalized to β‐actin housekeeping gene. Data are represented as mean ± S.E.M. *N *=* *3 in each group *P <* *0.05, **P <* *0.01. (**B**) Immunofluorescence staining for sFlt1 and PIGF expression in HTR‐8/SVneo cells in the presence of C5a or PBS (CON). Scale bar: 60 μm.

Next, we detected the effects of C5a on regulating angiogenic‐related factors expression in trophoblast cells. C5a significantly elevated the mRNA levels of anti‐angiogenic factors such as IL‐1β, TNF‐α, IL‐6, MCP‐1 and sFlt1, but reduced the expression of pro‐angiogenic factors such as PIGF and IL‐10 in HTR‐8/SVneo cells (Fig. 4A). Immunofluorescent analysis further confirmed increasing of sFlt1 and decreasing of PIGF in HTR‐8/SVneo cells upon C5a stimulation (Fig. 4B).

### Effect of C5a on trophoblasts proliferation, migration and capillary‐like tube formation

**Figure 5:**
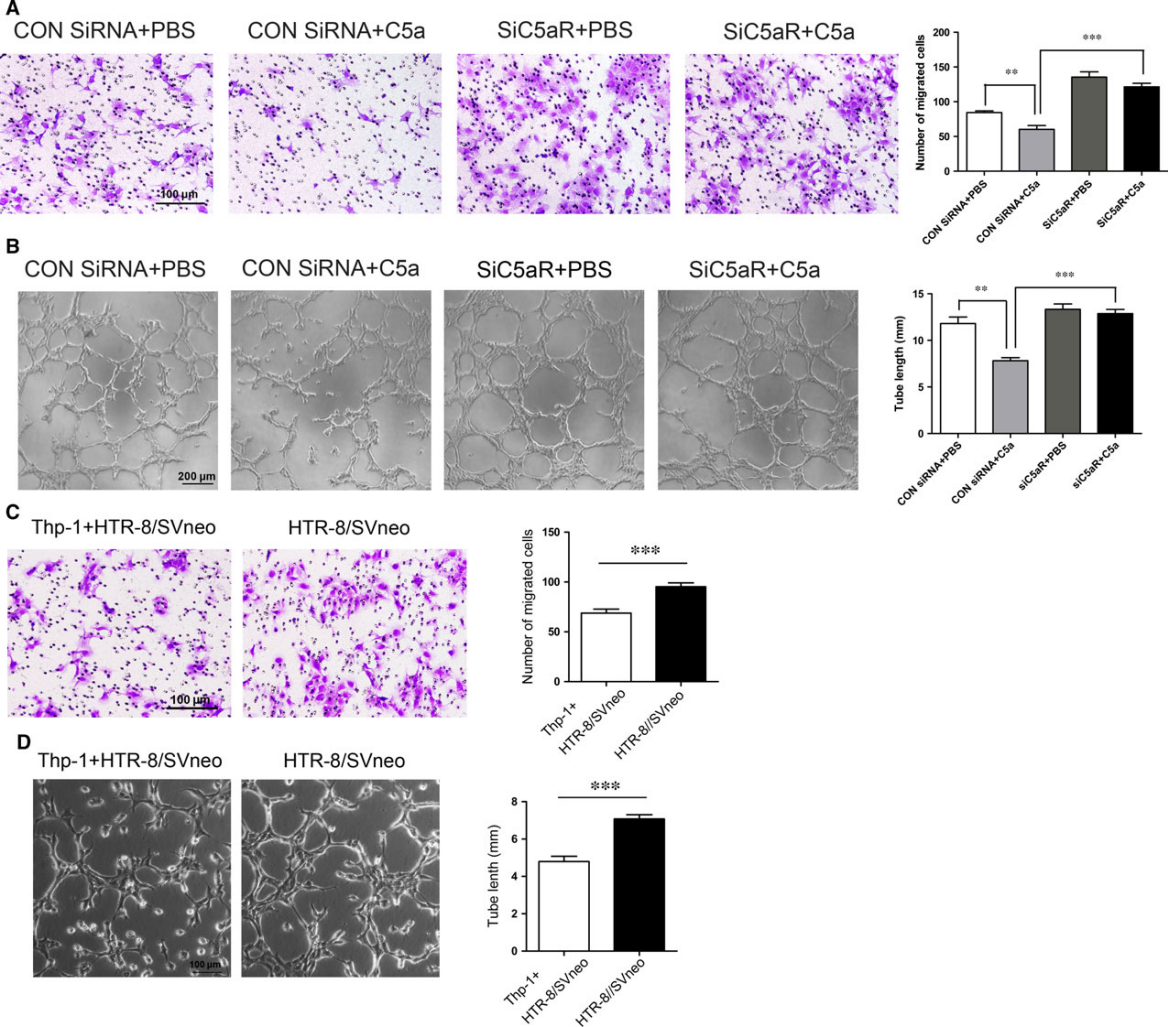
C5a/C5aR axis inhibited the migration and tube formation of trophoblast cells in HTR‐8/SVneo cells. HTR‐8/Svneo cells were transfected with C5aR SiRNA (SiC5aR) or Control SiRNA (CON SiRNA) and treated with C5a or PBS. (**A**) Transwell assay was used to evaluate the migration capacity of HTR‐8/SVneo cells. Scale bar = 100 μm. Bar graphs showed quantification of migrated cell numbers. **P <* *0.01, ***P <* *0.001. Data are represented as mean ± S.E.M. (**B**) Tube formation assay was conducted to analyse the ability of HTR‐8/SVneo cells to form capillary‐like structures. Representative photomicrographs were taken, and bar graphs showed quantification of tubule lengths. Data are presented as mean ± S.E.M.; **P <* *0.01; ***P <* *0.001. (**C** and **D**), HTR‐8/SVneo cells were co‐cultured with macrophages (Thp‐1 cells). Migration and tube formation abilities of HTR‐8/SVneo cells were significantly inhibited. Bar graphs showed quantification of migrated cell numbers. Data are presented as mean ± S.E.M.; ***P <* *0.001.

To detect the role of the C5a/C5aR axis in trophoblasts proliferation, migration and differentiation, HTR‐8/SVneo trophoblast cells were transfected with C5aR siRNA (siC5aR) or control siRNA, and the knockdown efficiency was assessed by Western blot and qPCR (Fig. S3). C5a had no significant effects on trophoblast proliferation as assessed by CCK‐8 assay (Fig. S4). The transwell assay was used to evaluate trophoblast migration. C5a (100 nM) dramatically reduced the migration of HTR‐8/SVneo cells, while siC5aR‐transfection rescued the trophoblast migration (Fig. 5A). In addition, C5a (100 nM) stimulation resulted in a significant attenuation of HTR‐8/SVneo tube formation, with a decrease in total sprout length in matrigel assay. In contrast, cells transfected with siC5aR showed high levels of capillary‐like network formation (Fig. 5B). We also detected the effect of macrophages (thp‐1 cells) on the migration and tube formation ability of HTR‐8/SVneo cells, with result showing that macrophages could block the migration and tube formation ability of trophoblasts (Fig. 5C, D). These data suggested that the C5a/C5aR axis is involved in the anti‐angiogenesis of trophoblasts.

### Clinical characteristics of the study population

The clinical and laboratory characteristics are presented in Table 1. Compared to the normal controls, women with PE were more likely to deliver smaller babies earlier. Levels of creatinine, high‐density lipoprotein cholesterol and C5a were significantly increased in women with PE compared to normal controls (*P *<* *0.05). Additionally, we investigated the longitudinal changes in serum C5a levels in 10 women with PE. Compared to 1 month before delivery, C5a levels were significantly higher 3 days after delivery, and then reduced to a lower level 3 months after delivery (Fig. S5). Importantly, univariate and multivariate logistic regression analyses indicated that C5a and creatinine were recognized as independent prognostic factors in patients with PE (Table 2).

**Table 1:** Clinical and laboratory characteristics of the study population

	Normal pregnant group (*n *=* *32)	PE group (*n *=* *24)	*P* Value
Maternal age, years	29.2 ± 0.7	30.7 ± 0.9	0.18
Gestational weeks at entry, week	28.7 ± 0.7	28.6 ± 0.8	0.91
Gestational weeks at delivery, week	39.3 ± 0.2	33.3 ± 0.7	<0.0001
BMI, kg/m^2^	21.6 ± 0.6	23.4 ± 0.7	0.06
Nulliparity, *n* (%)	27 (84.4)	20 (83.3)	>1.0
Systolic blood pressure, mmHg	120.2 ± 1.2	161.4 ± 4.2	<0.0001
Diastolic blood pressure, mmHg	75.4 ± 1.0	100.1 ± 2.4	<0.0001
Proteinuria, g/24 hrs	–	5.5 ± 0.9	–
Birthweight, g	3415 ± 70.2	1871 ± 193.9	<0.0001
Creatinine, μmol/l	40.2 ± 1.3	49.4 ± 2.0	0.0002
TC, mmol/l	5.4 ± 0.2	5.9 ± 1.2	0.06
TG, mmol/l	2.0 ± 0.1	3.1 ± 0.6	0.05
HDL‐C, mmol/l	2.1 ± 0.1	1.7 ± 0.1	0.0002
LDL‐C, mmol/l	2.7 ± 0.1	3.0 ± 0.2	0.11
C5a, ng/ml	76.4 ± 3.0	100.4 ± 5.9	0.001
C3a, μg/ml	25. 9 ± 2.1	30.1 ± 3.3	0.28

BMI, body mass index; TC, total cholesterol; TG, triglyceride; HDL‐C, high‐density lipoprotein cholesterol; LDL‐C, low‐density lipoprotein cholesterol.

Values are presented as mean ± S.E.M. or median (interquartile range).

**Table 2:** The risk factors of pre‐eclampsia in simple logistic regression

	Univariant			Multivariate		
	OR	95%CI	*P*	OR	95%CI	*P*
Maternal age, years	1.093	0.959–1.245	0.184			
BMI, kg/m^2^	1.165	0.987–1.376	0.072			
C5a, ng/ml	1.052	1.018–1.086	0.002	1.056	1.014–1.099	0.0009
C3a, μg/ml	1.023	0.982–1.065	0.279			
Creatinine, μmol/l	1.128	1.045–1.217	0.002	1.106	1.014–1.207	0.023
TC, mmol/l	1.669	0.879–3.172	1.118			
TG, mmol/l	1.777	0.980–3.221	0.058			
HDL‐C, mmol/l	0.066	0.013–0.347	0.001	0.101	0.016–0.646	0.101
LDL‐C, mmol/l	1.669	0.879–3.172	0.118			

BMI, body mass index; TC, total cholesterol; TG, triglyceride; HDL‐C, high‐density lipoprotein cholesterol; LDL‐C, low‐density lipoprotein cholesterol.

### Maternal vascular characteristics of the study population

**Figure 6:**
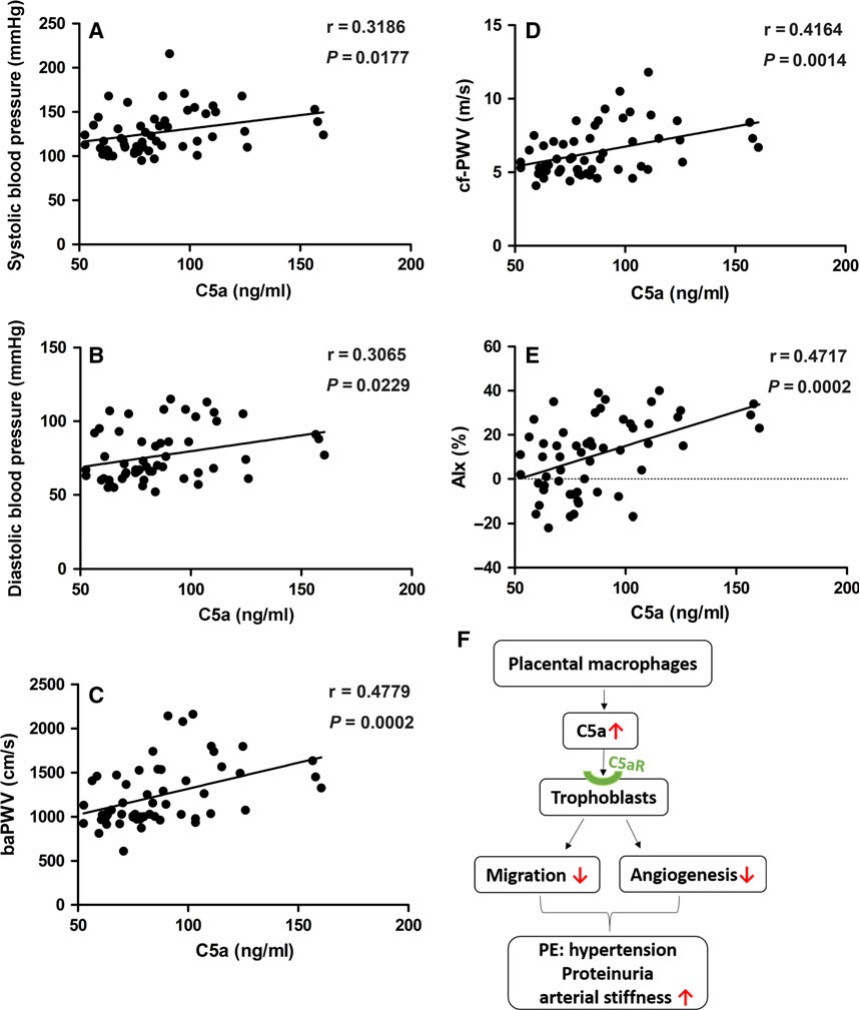
Correlation between maternal serum level of C5a and measures of arterial stiffness. The relationship between maternal serum levels of C5a and systolic blood pressure (**A**), diastolic blood pressure (**B**), ba‐PWV (**C**), cf‐PWV (**D**) and AIx (**E**). The linear regression line is shown in case of a significant correlation. (**F**) Summary of the role of C5a in the pathogenesis of PE.

The vascular characteristics of study population are given in Table 3. The peripheral and central systolic blood pressure, diastolic blood pressure, pulse pressures, mean arterial blood pressure, AIx, AIx@75HR (AIx at heart rate of 75/min.) and PWVs (carotid–femoral and brachial–ankle) were significantly higher in women with PE compared to normal controls. We next explored the relationship between maternal circulating C5a and blood pressure and arterial stiffness using Pearson correlation analysis. The results revealed a significant positive correlation between maternal serum C5a concentration and systolic blood pressure, diastolic blood pressure, ba‐PWV, cf‐PWV and AIx, respectively (Fig. 6). However, we use multiple linear regression to explore the relation between C5a and all these factors and the result showed that the coefficients between C5a and all these factors have no statistical significance (Table S2).

**Table 3:** Vascular characteristics of study populations

	Normal pregnant group (*n *=* *32)	PE group (*n *=* *24)	*P* Value
Heart rate, bmp	83.8 ± 1.9	78.5 ± 2.0	0.077
Peripheral SBP, mmHg	110.3 ± 1.5	151.0 ± 4.2	<0.0001
Peripheral DBP, mmHg	64.0 ± 1.1	92.9 ± 2.5	<0.0001
MAP, mmHg	79.5 ± 1.1	112.3 ± 2.9	<0.0001
Peripheral PP, mmHg	46.5 ± 1.0	58.2 ± 2.7	0.0006
Central SBP, mmHg	93.1 ± 1.4	137.9 ± 4.5	<0.0001
Central DBP, mmHg	65.7 ± 1.2	93.5 ± 2.4	<0.0001
Central PP, mmHg	27.4 ± 0.9	42.4 ± 2.9	<0.0001
AIx, %	−0.3 ± 2.1	25.6 ± 1.9	<0.0001
AIx@HR75, %	3.8 ± 2.1	27.5 ± 1.7	<0.0001
cf‐PWV, m/sec.	5.3 ± 0.1	7.7 ± 0.3	<0.0001
ba‐PWV, cm/sec.	997.6 ± 19.7	1556 ± 59.4	<0.0001

Values are mean ± S.E.M.

SBP, systolic blood pressure; DBP, diastolic blood pressure; MAP, mean arterial pressure; PP, pulse pressure; AIx, augmentation index; AIx@HR75; AIx at heart rate of 75/min.; cf‐PWV, carotid–femoral pulse wave velocity; ba‐PWV, brachial–ankle pulse wave velocity.

## Discussion

Our findings indicated that activation of C5a in placenta could regulate the function of trophoblast cells. Mechanically, placental C5a interact with its receptor C5aR, interrupts trophoblasts function *via* inhibiting the migration, tube formation and angiogenesis of trophoblasts. Additionally, increased serum levels of C5a were well correlated with maternal blood pressure and arterial stiffness.

The complement system includes a variety of complement components and some of them have been reported to be associated with PE 22, 23, 24, 25, 26. Upon activation of the complement cascade, the formation of C3 and C5 convertase results in the cleavage of the complement component C5 to C5a and C5b. As we know, eculizumab is a monoclonal antibody inhibitor of C5, preventing its cleavage to C5a and C5b. Burwick and Feinberg reported the case that eculizumab treatment effectively reduced haemolysis and normalized platelet counts in one woman with HELLP syndrome and prolonged the pregnancy by 17 days 27. In addition, they confirmed that urinary marker C5b‐9 correlated strongly with the anti‐angiogenic factors. Urinary C5a and C5b‐9 may be more specific to severe PE and more useful in guiding response to eculizumab 28, 29. However, further *in vivo* studies are necessary. In the non‐pregnant state, C5 is predominantly secreted by the liver, and then C5a activation occurs and consequently elicits a broad range of biological functions by activating different cell types such as neutrophils, monocytes, vascular smooth muscle cells and cardiomyocytes 30. Whereas, our study is the first to explore the effect of C5a on trophoblast cells, which are only found in the placenta. We confirmed that the enhanced C5a levels were mainly derived from infiltrated placental CD11b^+^ macrophages. Taken together, we provide the evidence that activation of macrophages could exert a negative effect on trophoblast function.

As is well known, insufficient spiral arterial remodelling causes abnormal placentation, and inadequate placental perfusion is a central stage in the onset of PE 6. Trophoblasts are the major component of the placenta tissue, presenting a placenta‐specific property. Defective trophoblasts proliferation, migration/invasion accompanied by poor angiogenesis, are involved in poor spiral arterial remodelling. Previous study 31 reported that C5aR antagonist attenuated placental ischaemia‐induced hypertension and followed endothelial dysfunction in rat reduced uterine perfusion pressure (RUPP) model, but the mechanism was still unknown. Here, we provided direct evidence that C5a/C5aR axis exerts an inhibitory role in regulating the trophoblasts function, which could be involved in the spiral arterial remodelling. Our data demonstrated that C5a inhibited the migration and tube formation abilities of trophoblast cells *via* its receptor C5aR. C5a is also reported to be an angiogenic factor, which exerts a crucial role in stimulating macrophages towards an angiogenesis‐inhibitory phenotype in the model of retinopathy of prematurity 32. In contrast, a pro‐angiogenic effect of C5a was observed on endothelium cells in age‐related macular degeneration and lung cancer mouse model 33, 34. Whereas our study suggested that trophoblasts displayed an anti‐angiogenic phenotype when stimulated by C5a. Taken together, the various effects of C5a on angiogensis are influenced by pathophysiological states and tissue microenvironment. Previous studies have demonstrated that the imbalance of pro‐angiogenic factor PIGF and the anti‐angiogenic factor sFlt1 is involved in the development of PE 35, 36, 37. In the present study, we confirmed that C5a reduced the expression of PIGF and simultaneously increased expression of sFlt1 in trophoblast cells. Besides, C5a had a similar effect on expression of IL‐6, IL‐10 and TNF‐α in trophoblasts, which were regarded as angiogenic factors as well 32. The severe poor placental growth would ultimately lead to clinical manifestations including hypertension and proteinuria. PE is a systemic vascular disorder characterized by widespread maternal endothelial dysfunction 38. Of note, increased arterial stiffness is associated with an increased risk of cardiovascular morbidity and mortality 39, 40. Arterial stiffness is a key determinant of central aortic pressure and reflects the early structural and functional damage of the vascular wall. PWVs and AIx are useful indexes for evaluating arterial stiffness, and cf‐PWV is considered the ‘gold standard’ measurement for the stiffness of the aorta. Previous studies have shown that normal pregnancy is associated with a decrease in maternal blood pressure, AIx and PWV value in the second trimester 21, 41, and then the physiological decrease gradually reverts to pre‐pregnancy levels. Some studies have reported increased arterial stiffness in women with PE, as measured by cf‐PWV, AIx and other indexes. However, Anastasakis *et al*. 42 found no significant association between cf‐PWV and PE, although the small sample size (*n *=* *6) may reduce the reliability of the result. Several longitudinal studies suggested the predictive value of arterial stiffness measurements for the onset of PE 21, 43. Notably, our findings revealed that women with PE suffer poor vascular function during their pregnancy with enhanced maternal peripheral and central pressures and arterial stiffness. Consistent with this, systemic activation of C5a was observed in women with PE. However, C5a levels decreased when women recover from PE. We and others have demonstrated that C5a was related to vascular injury in hypertensive animal models 44, 45. Of interest, our data suggest that maternal serum levels of C5a were positively correlated with systolic and diastolic blood pressure, ba‐PWV, cf‐PWV and AIx, respectively. It indicated that C5a was associated with maternal vascular dysfunction in PE. Further study is needed to support the perspective.

In conclusion, excessive complement activation is involved in the dysfunction of trophoblasts. Besides its pro‐inflammatory effect, C5a directly regulates the migration and angiogenesis of trophoblasts and is also associated with maternal blood pressure and arterial stiffness. Overall, our study provides a novel insight into the possibility that potential agents targeting the C5a/C5aR axis may prevent the development of PE without disturbing the remainder of the complement cascade for host defence.

## Conflict of interest

The authors confirm that there is no conflict of interests.

## Supporting information


**Table S1** Sequences of PCR primers.
**Table S2** Analysis of multiple linear regression for C5a based on ba‐PWV, cf‐WV, AIx, systolic and diastolic blood pressure.
**Fig. S1** Expression of C5a in placentas of early and late pregnancy.
**Fig. S2** Expression of C5a and C5aR in macrophages and trophoblasts.
**Fig. S3** C5aR protein and mRNA expression in siRNA treated HTR8/SVneo cells.
**Fig. S4** C5a has no effect on trophoblast cells proliferation.
**Fig. S5** Longitudinal changes in serum C5a levels of women PE.

## References

[1] Report of the National High Blood Pressure Education . Program working group on high blood pressure in pregnancy. Am J Obstet Gynecol. 2000; 183: S1–22. 10920346

[2] Sibai B , Dekker G , Kupferminc M . Pre‐eclampsia. Lancet. 2005; 365: 785–99. 15733721 10.1016/S0140-6736(05)17987-2

[3] Brown DW , Dueker N , Jamieson DJ , *et al* Preeclampsia and the risk of ischemic stroke among young women: results from the stroke prevention in young women study. Stroke. 2006; 37: 1055–9. 16484606 10.1161/01.STR.0000206284.96739.ee

[4] McDonald SD , Malinowski A , Zhou Q , *et al* Cardiovascular sequelae of preeclampsia/eclampsia: a systematic review and meta‐analyses. Am Heart J. 2008; 156: 918–30. 19061708 10.1016/j.ahj.2008.06.042

[5] Lykke JA , Langhoff‐Roos J , Sibai BM , *et al* Hypertensive pregnancy disorders and subsequent cardiovascular morbidity and type 2 diabetes mellitus in the mother. Hypertension. 2009; 53: 944–51. 19433776 10.1161/HYPERTENSIONAHA.109.130765

[6] Sargent IL , Borzychowski AM , Redman CW . Immunoregulation in normal pregnancy and pre‐eclampsia: an overview. Reprod Biomed Online. 2006; 13: 680–6. 17169180 10.1016/s1472-6483(10)60659-1

[7] Kaufmann P , Black S , Huppertz B . Endovascular trophoblast invasion: implications for the pathogenesis of intrauterine growth retardation and preeclampsia. Biol Reprod. 2003; 69: 1–7. 12620937 10.1095/biolreprod.102.014977

[8] Brosens IA , Robertson WB , Dixon HG . The role of the spiral arteries in the pathogenesis of preeclampsia. Obstet Gynecol Annu. 1972; 1: 177–91. 4669123

[9] Roberts JM , Gammill HS . Preeclampsia: recent insights. Hypertension. 2005; 46: 1243–9. 16230510 10.1161/01.HYP.0000188408.49896.c5

[10] Tincani A , Bazzani C , Zingarelli S , *et al* Lupus and the antiphospholipid syndrome in pregnancy and obstetrics: clinical characteristics, diagnosis, pathogenesis, and treatment. Semin Thromb Hemost. 2008; 34: 267–73. 18720306 10.1055/s-0028-1082270

[11] Chakravarty EF , Nelson L , Krishnan E . Obstetric hospitalizations in the United States for women with systemic lupus erythematosus and rheumatoid arthritis. Arthritis Rheum. 2006; 54: 899–907. 16508972 10.1002/art.21663

[12] Guo RF , Ward PA . Role of C5a in inflammatory responses. Annu Rev Immunol. 2005; 23: 821–52. 15771587 10.1146/annurev.immunol.23.021704.115835

[13] Singh J , Ahmed A , Girardi G . Role of complement component C1q in the onset of preeclampsia in mice. Hypertension. 2011; 58: 716–24. 21859968 10.1161/HYPERTENSIONAHA.111.175919

[14] Wang W , Irani RA , Zhang Y , *et al* Autoantibody‐mediated complement C3a receptor activation contributes to the pathogenesis of preeclampsia. Hypertension. 2012; 60: 712–21. 22868393 10.1161/HYPERTENSIONAHA.112.191817 PMC4131740

[15] Lynch AM , Salmon JE . Dysregulated complement activation as a common pathway of injury in preeclampsia and other pregnancy complications. Placenta. 2010; 31: 561–7. 20427084 10.1016/j.placenta.2010.03.010 PMC2900404

[16] Ruan CC , Ge Q , Li Y , *et al* Complement‐mediated macrophage polarization in perivascular adipose tissue contributes to vascular injury in deoxycorticosterone acetate‐salt mice. Arterioscler Thromb Vasc Biol. 2015; 35: 598–606. 25573852 10.1161/ATVBAHA.114.304927

[17] Denny KJ , Coulthard LG , Finnell RH , *et al* Elevated complement factor C5a in maternal and umbilical cord plasma in preeclampsia. J Reprod Immunol. 2013; 97: 211–6. 23415845 10.1016/j.jri.2012.11.006

[18] American College of O, Gynecologists, Task Force on Hypertension in P . Hypertension in pregnancy. Report of the American college of obstetricians and gynecologists’ task force on hypertension in pregnancy. Obstet Gynecol. 2013; 122: 1122–31. 24150027 10.1097/01.AOG.0000437382.03963.88

[19] Huang QF , Sheng CS , Liu M , *et al* Arterial stiffness and wave reflections in relation to plasma advanced glycation end products in a Chinese population. Am J Hypertens. 2013; 26: 754–61. 23449605 10.1093/ajh/hpt014

[20] Sheng CS , Li Y , Li LH , *et al* Brachial‐ankle pulse wave velocity as a predictor of mortality in elderly Chinese. Hypertension. 2014; 64: 1124–30. 25259749 10.1161/HYPERTENSIONAHA.114.04063

[21] Oyama‐Kato M , Ohmichi M , Takahashi K , *et al* Change in pulse wave velocity throughout normal pregnancy and its value in predicting pregnancy‐induced hypertension: a longitudinal study. Am J Obstet Gynecol. 2006; 195: 464–9. 16647682 10.1016/j.ajog.2006.01.104

[22] Lynch AM , Murphy JR , Byers T , *et al* Alternative complement pathway activation fragment Bb in early pregnancy as a predictor of preeclampsia. Am J Obstet Gynecol. 2008; 198(385): e1–9. 10.1016/j.ajog.2007.10.793 PMC2362503 18221926

[23] Buurma A , Cohen D , Veraar K , *et al* Preeclampsia is characterized by placental complement dysregulation. Hypertension. 2012; 60: 1332–7. 23006730 10.1161/HYPERTENSIONAHA.112.194324

[24] Derzsy Z , Prohaszka Z , Rigo J Jr , *et al* Activation of the complement system in normal pregnancy and preeclampsia. Mol Immunol. 2010; 47: 1500–6. 20181396 10.1016/j.molimm.2010.01.021

[25] Penning M , Chua JS , van Kooten C , *et al* Classical complement pathway activation in the kidneys of women with preeclampsia. Hypertension. 2015; 66: 117–25. 25941343 10.1161/HYPERTENSIONAHA.115.05484 PMC4465860

[26] Lokki AI , Heikkinen‐Eloranta J , Jarva H , *et al* Complement activation and regulation in preeclamptic placenta. Front Immunol. 2014; 5: 312. 25071773 10.3389/fimmu.2014.00312 PMC4088925

[27] Burwick RM , Feinberg BB . Eculizumab for the treatment of preeclampsia/HELLP syndrome. Placenta. 2013; 34: 201–3. 23228435 10.1016/j.placenta.2012.11.014

[28] Burwick RM , Fichorova RN , Dawood HY , *et al* Urinary excretion of C5b‐9 in severe preeclampsia: tipping the balance of complement activation in pregnancy. Hypertension. 2013; 62: 1040–5. 24060886 10.1161/HYPERTENSIONAHA.113.01420

[29] Guseh SH , Feinberg BB , Dawood HY , *et al* Urinary excretion of C5b‐9 is associated with the anti‐angiogenic state in severe preeclampsia. Am J Reprod Immunol. 2015; 73: 437–44. 25521546 10.1111/aji.12349

[30] Manthey HD , Woodruff TM , Taylor SM , *et al* Complement component 5a (C5a). Int J Biochem Cell Biol. 2009; 41: 2114–7. 19464229 10.1016/j.biocel.2009.04.005

[31] Lillegard KE , Loeks‐Johnson AC , Opacich JW , *et al* Differential effects of complement activation products c3a and c5a on cardiovascular function in hypertensive pregnant rats. J Pharmacol Exp Ther. 2014; 351: 344–51. 25150279 10.1124/jpet.114.218123 PMC4201271

[32] Langer HF , Chung KJ , Orlova VV , *et al* Complement‐mediated inhibition of neovascularization reveals a point of convergence between innate immunity and angiogenesis. Blood. 2010; 116: 4395–403. 20625009 10.1182/blood-2010-01-261503 PMC2996109

[33] Nozaki M , Raisler BJ , Sakurai E , *et al* Drusen complement components C3a and C5a promote choroidal neovascularization. Proc Natl Acad Sci USA. 2006; 103: 2328–33. 16452172 10.1073/pnas.0408835103 PMC1413680

[34] Corrales L , Ajona D , Rafail S , *et al* Anaphylatoxin C5a creates a favorable microenvironment for lung cancer progression. J Immunol. 2012; 189: 4674–83. 23028051 10.4049/jimmunol.1201654 PMC3478398

[35] Spradley FT , Tan AY , Joo WS , *et al* Placental growth factor administration abolishes placental ischemia‐induced hypertension. Hypertension. 2016; 67: 740–7. 26831193 10.1161/HYPERTENSIONAHA.115.06783 PMC4786447

[36] LaMarca B , Amaral LM , Harmon AC , *et al* Placental ischemia and resultant phenotype in animal models of preeclampsia. Curr Hypertens Rep. 2016; 18: 38. 27076345 10.1007/s11906-016-0633-x PMC5127437

[37] Wu FT , Stefanini MO , Mac Gabhann F , *et al* A systems biology perspective on sVEGFR1: its biological function, pathogenic role and therapeutic use. J Cell Mol Med. 2010; 14: 528–52. 19840194 10.1111/j.1582-4934.2009.00941.x PMC3039304

[38] Powe CE , Levine RJ , Karumanchi SA . Preeclampsia, a disease of the maternal endothelium: the role of antiangiogenic factors and implications for later cardiovascular disease. Circulation. 2011; 123: 2856–69. 21690502 10.1161/CIRCULATIONAHA.109.853127 PMC3148781

[39] Mattace‐Raso FU , van der Cammen TJ , Hofman A , *et al* Arterial stiffness and risk of coronary heart disease and stroke: the Rotterdam Study. Circulation. 2006; 113: 657–63. 16461838 10.1161/CIRCULATIONAHA.105.555235

[40] Vlachopoulos C , Aznaouridis K , Stefanadis C . Prediction of cardiovascular events and all‐cause mortality with arterial stiffness: a systematic review and meta‐analysis. J Am Coll Cardiol. 2010; 55: 1318–27. 20338492 10.1016/j.jacc.2009.10.061

[41] Macedo ML , Luminoso D , Savvidou MD , *et al* Maternal wave reflections and arterial stiffness in normal pregnancy as assessed by applanation tonometry. Hypertension. 2008; 51: 1047–51. 18259025 10.1161/HYPERTENSIONAHA.107.106062

[42] Anastasakis E , Paraskevas KI , Papantoniou N , *et al* Association between abnormal uterine artery Doppler flow velocimetry, risk of preeclampsia, and indices of arterial structure and function: a pilot study. Angiology. 2008; 59: 493–9. 18504265 10.1177/0003319708316008

[43] Robb AO , Mills NL , Din JN , *et al* Influence of the menstrual cycle, pregnancy, and preeclampsia on arterial stiffness. Hypertension. 2009; 53: 952–8. 19398652 10.1161/HYPERTENSIONAHA.109.130898

[44] Ruan CC , Ma Y , Ge Q , *et al* Complement‐mediated inhibition of adiponectin regulates perivascular inflammation and vascular injury in hypertension. FASEB J. 2017; 31: 1120–9. 27974594 10.1096/fj.201600780R

[45] Iyer A , Woodruff TM , Wu MC , *et al* Inhibition of inflammation and fibrosis by a complement C5a receptor antagonist in DOCA‐salt hypertensive rats. J Cardiovasc Pharmacol. 2011; 58: 479–86. 21753735 10.1097/FJC.0b013e31822a7a09

