# Supplementary material for: Complement 5a‐mediated trophoblasts dysfunction is involved in the development of pre‐eclampsia
Source: J Cell Mol Med. 2017 Nov 23;22(2):1034–46. doi: 10.1111/jcmm.13466 (PMC5783881; doi:10.1111/jcmm.13466)
Supplement: Supplementary file 1 — Table S1 Sequences of PCR primers. Table S2 Analysis of multiple linear regression for C5a based on ba‐PWV, cf‐WV, AIx, systolic and diastolic blood pressure. Fig. S1 Expression of C5a in placentas of early and late pregnancy. Fig. S2 Expression of C5a and C5aR in macrophages and trophoblasts. Fig. S3 C5aR protein and mRNA expression in siRNA treated HTR8/SVneo cells. Fig. S4 C5a has no effect on trophoblast cells proliferation. Fig. S5 Longitudinal changes in serum C5a levels of women PE. [file JCMM-22-1034-s001.docx]

**Table S1.** Sequences of PCR primers

|  | Forward primer sequence | Reverse primer sequence |
| --- | --- | --- |
| IL-6 | 5'AAGCCAGAGCTGTGCAGATGAGTA | 5' TGTCCTGCAGCCACTGGTTC |
| IL-10 | 5'GAGATGCCTTCAGCAGAGTGAAGA | 5'AGGCTTGGCAACCCAGGTAAC |
| TNF-α | 5' CAGAGGGCCTGTACCTCATC | 5' GGAAGACCCCTCCCAGATAG |
| MCP-1 | 5' TCTGTGCCTGCTGCTCATAG | 5' CTTGGGTTGTGGAGTGAGTG |
| IL-8 | 5' GGGCCATCAGTTGCAAATC | 5' GCTTGTGTGCTCTGCTGTCTC |
| sFlt1 | 5' TAATCATTCCGAAGCAAGG | 5' AGAGTCAGCCACAACCAAG |
| PIGF | 5' CTGTGCCTTGCTTATGTTTGT | 5' CTGTCATCTGCCATGGTCTTT |
| C3 | 5' AAGTGGTGGAGAAGGTGGT | 5' GATCCGATAGAGAACTGTGG |
| C5 | 5' TGCCCTACCTGATTCTCTAA | 5' TGAACACCTTTGGCTTGAC |
| C5aR | 5' TGCTGACCATACCCTCCTTC | 5' CCCTAACCACGGACTCTTCA |
| β-actin | 5' CTCCATCCTGGCCTCGCTGT | 5' GCTGTCACCTTCACCGTTCC |

IL, interleukin; TNF-α: tumor necrosis factor-α; MCP-1, monocyte Chemoattractant Protein-1; sFlt1, soluble fms-like tyrosine kinase 1; PIGF, placental growth factor; C5aR, C5a receptor.


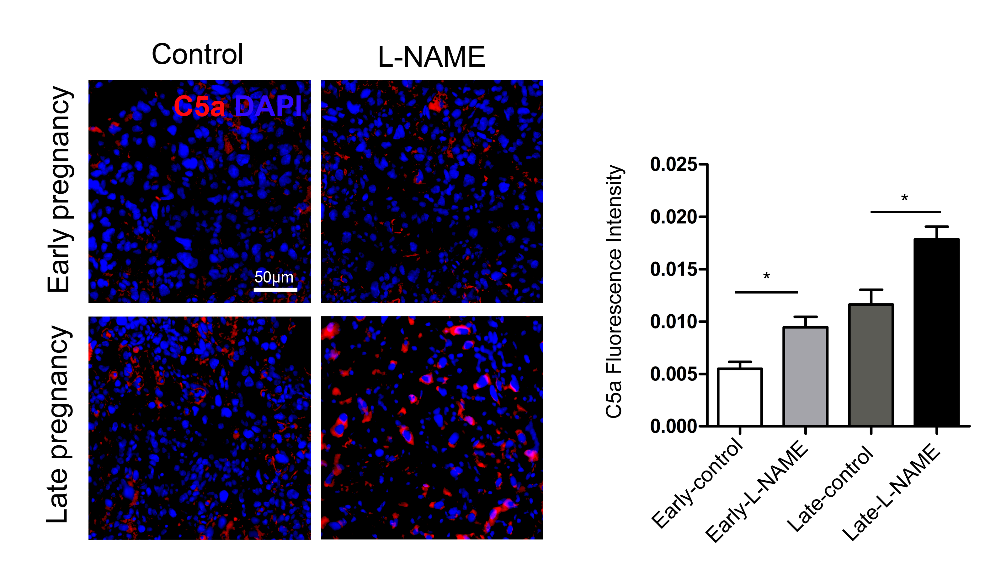


**Figure. S1 Expression of C5a in placentas of early and late pregnancy**

The expression of C5a in early (gestational day 12) and late (gestational day 20) pregnancy in L-NAME induced preeclampsia mice model. C5a level was increased in the placenta of L-NAME-induced PE mice during early pregnancy. In late pregnancy, C5a level was further enhanced in placentas of PE mice. The right panel showed the statistical analysis. N=3 in each group. Data are shown as mean±SEM. **P*<0.05.


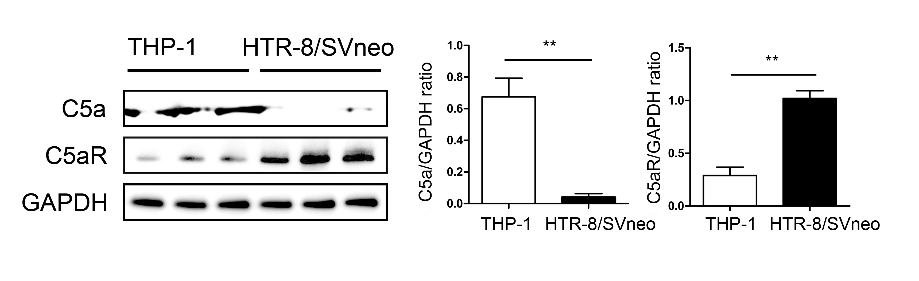


**Figure. S2 Expression of C5a and C5aR in macrophages and trophoblasts**

C5a was mainly expressed in macrophages (THP-1 cells) and C5aR was expressed in both macrophages and trophoblasts (HTR-8/SVneo cells), especially in trophoblasts.

The right panel showed the statistical analysis. N=6 in each group. Data are shown as mean±SEM. ***P*<0.01.

**
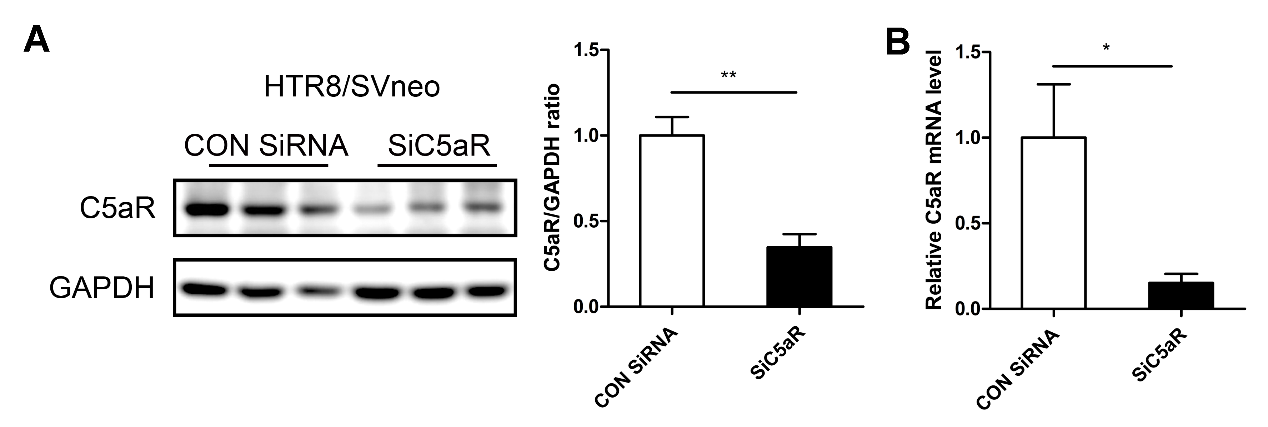
**

**Figure. S3 C5aR protein and mRNA expression in siRNA treated HTR8/SVneo cells.**

(**A**) Western blot analysis of C5aR in HTR8/SVneo cells transfected with control siRNA or siC5aR. GAPDH was used to normalize for loading variability. The ratio of C5aR to GAPDH protein levels was significantly decreased in HTR8/SVneo cells transfected with siC5aR compared to control siRNA. (**B**) C5aR mRNA level of HTR8/SVneo cells transfected with siC5aR was significantly decreased compared to control siRNA. The C5aR mRNA was normalized to β-actin housekeeping gene. Data are presented as mean ± SEM. **P*<0.05, ***P*<0.01.


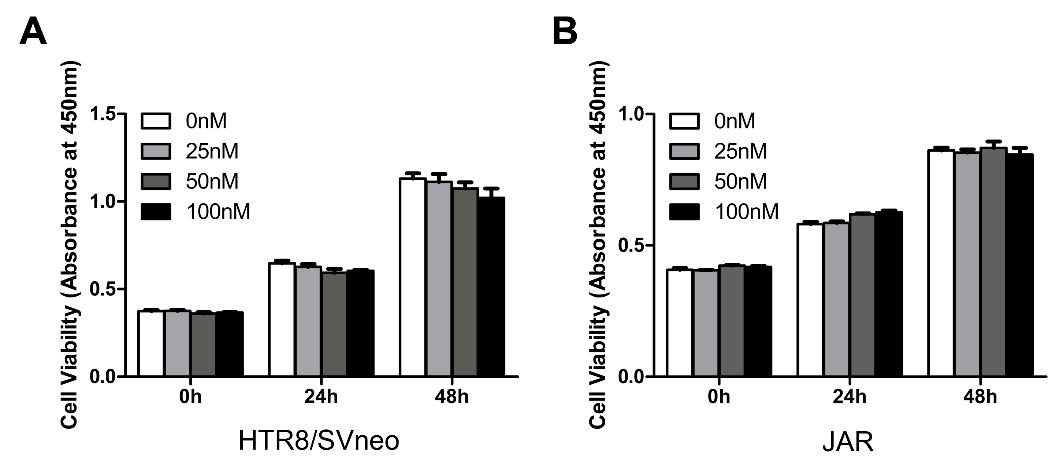


**Figure S4. C5a has no effect on trophoblast cells proliferation.**

Proliferation of HTR8/SVneo cells was performed in the presence of wide range of C5a concentrations (0, 25, 50, 100nM) for 24 or 48h. No effect of C5a on trophoblast cells was detected. Cell viability was evaluated by CCK8 assay. Data are presented as mean ± SEM.


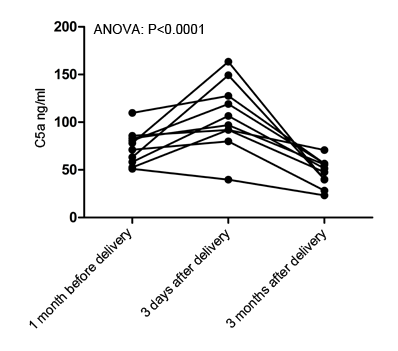


**Figure S5. Longitudinal changes in serum C5a levels of women PE.**

Longitudinal changes in serum levels of C5a at 1 month before, 3 days and 3 months after delivery in 10 preeclamptic women.

**Table S2**. Analysis of multiple linear regression for C5a based on ba-PWV, cf-WV, AIx, systolic and diastolic blood pressure.

| Variable | β | 95% CI | *P* value |
| --- | --- | --- | --- |
| ba-PWV | 0.015 | -0.253;0.056 | 0.455 |
| cf-WV | 1.424 | -6.172;9.021 | 0.708 |
| AIx | 0.483 | -0.087;1.054 | 0.095 |
| Systolic blood pressure | -0.116 | -0.882;0.649 | 0.761 |
| Diastolic blood pressure | 0.017 | -0.967;1.001 | 0.972 |

β, partial regression coefficients; 95% CI, 95% confidence interval.
